# Henri Rousseau—known as Le Douanier Rousseau (1844–1910). The Snake Charmer (1907).

**DOI:** 10.3201/eid0907.AC0907

**Published:** 2003-07

**Authors:** Polyxeni Potter

**Affiliations:** * Centers for Disease Control and Prevention, Atlanta, Georgia, USA

## Abstract

As the Gods began one world, and man another, So the snakecharmer begins a snaky sphere With moon-eye, mouth pipe. He pipes. Pipes green. Pipes water  –Sylvia Plath, "Snakecharmer"

**Figure:**
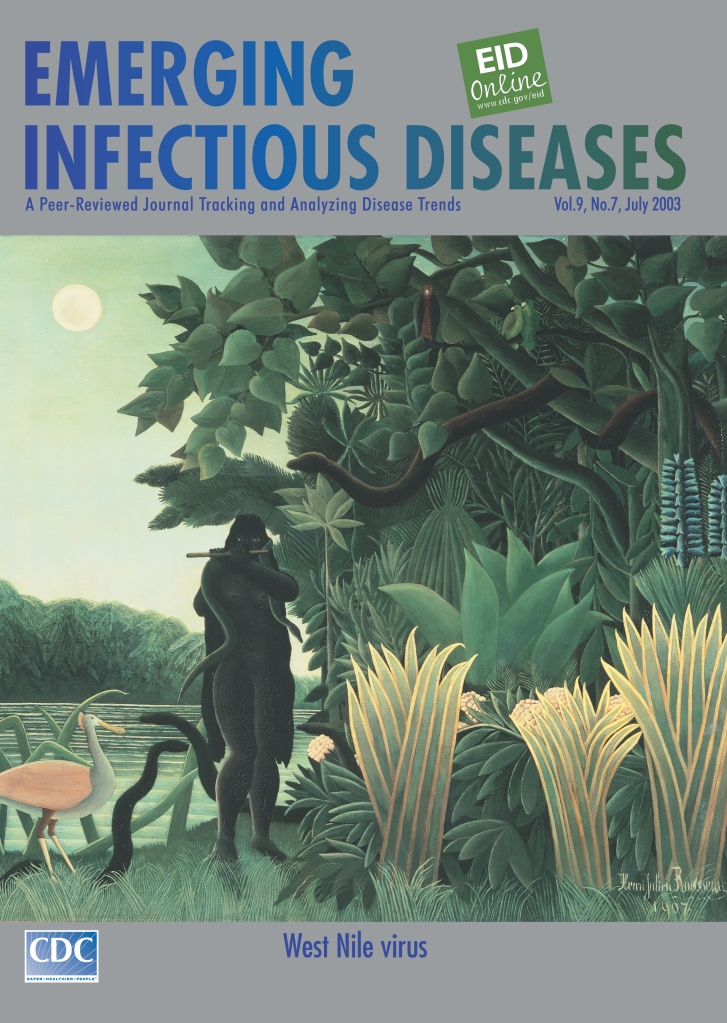
Oil on canvas, 169 cm x 189.5 cm. Musée d’Orsay, Paris, France. Credit: Réunion des Musées Nationaux/Art Resource, NY

Critics called him “naïve,” the term for painters with no formal training in art. Henri Rousseau, self-made late-bloomer from Laval, France, fit the definition. But his work proved that in art as in all ventures, training, though valuable, is not the key ingredient—not as key perhaps as talent, inspiration, or originality. Untrained in art but not uneducated by the standards of his day, Rousseau was a teacher and a military man. He was interested in politics and the realm of ideas. He knew music and poetry and even tried his hand as playwright. Dubbed “Le Douanier” (customs officer) after his main occupation outside art, he struggled in anonymity until near the end of his life, when he was discovered by Pablo Picasso and others and was recognized for his powerful individual style (*1*).

Like other naïve or primitive artists, Rousseau found art late in life. He took up painting as a hobby and soon retired from his job in the customs office to devote time to this new vocation. He copied the masters, struggled to learn their craft (particularly the academic style of Ingres), and aspired to paint like them. He exhibited often at the Salon des Indépendents in Paris, where artists could show their paintings without selection restrictions, and what he might have lost to technical clumsiness he seemed to make up in ingenuous charm. Although his work is difficult to categorize, he seems to have been influenced by his contemporary Paul Gauguin and his followers, the Nabis, who promoted directness of feeling and color harmony (*2*). Rousseau eventually found his own formal language and style, but what elevated his mature work to greatness were perhaps the very oblivion of convention, the freshness of approach, and the depth of discovery that comes from a truly unique perspective.

Rousseau’s exotic compositions owe nothing to traditional art methods yet defy modern labels. The fantastic vegetation in his jungle paintings (for which he is best known) has no equivalent in nature. These exotic landscapes, oversized and filled with exuberant color, were entirely imaginary. Although often inhabited by half-concealed wild beasts and laced with conflict, they exuded an eerie stillness. The images, smooth, vivid, and clearly defined, were flat and fluid against dense but dimensionless greenery, and although unreal and extraordinary, were rendered in meticulous botanical detail.

The Snake Charmer, on this month’s cover of Emerging Infectious Diseases, is one of Rousseau’s finest and most celebrated works. Like his other jungle paintings, it is filled with lush greenery. Punctuated by an uncoiling reptile at arm’s length, the thick vegetal screen that makes up most of the landscape is live with tension. The dark, undulating figure of the snake charmer dances ambiguously amidst a tangle of wildlife. Nature, framed by “a wave of flickering-grass tongues,” (*3*) looms in the foreground immediate and tangible, yet dreamlike and distant as the moon. In a trance, the animals are guided (it seems as much by the glossy stream as by the snake charmer’s reed) into a tight ecological web, where unbeknownst to them, they share more than the music.

Rousseau’s imagination, like that of many of his contemporaries in Paris, succumbed to the allure of exotic lands, where plants grew larger than life, wild animals held unknown powers and magnetism, and humans lounged in “Eden’s navel” (*3*) amidst all that was lost in the fall from grace. To explain the products of his inflamed imagination, Rousseau falsely claimed that he had visited Mexico. But unlike Gauguin, who went to Tahiti in search of inspiration, Rousseau traveled only vicariously and found his models in local gardens and the Paris zoo.

Exotic lands have become prosaic to us. What remains naïve and primitive is our knowledge of the forest’s architecture and the perils of its convergence with human habitat. But like the uncoiling snake in Rousseau’s painting, out of the impenetrable jungle comes knowledge about pestilences, piece by piece: a favorable environment, a stable population, a reservoir host, the agent. The emergence of West Nile virus in North America is a case in point. We, modern snake-charmers, must pipe the pieces (bird, horse, reptile) into a knowable, harmonious fabric.
